# Dietary Soy Preserves Cognitive Function in Experimental Fetal Alcohol Spectrum Disorder: Role of Increased Signaling through Notch and Gonadotropin Releasing Hormone Networks

**DOI:** 10.4236/jbbs.2025.152002

**Published:** 2025-02-26

**Authors:** Suzanne M. de la Monte, Ming Tong, Jason Ziplow, Princess Mark, Stephanie Van, Van Ahn Nguyen

**Affiliations:** 1 Departments of Pathology and Laboratory Medicine, Neurosurgery, and Neurology, Rhode Island Hospital, Providence, RI, USA; 2 Women & Infants Hospital, Brown University Health, Alpert Medical School of Brown University, Providence, Rhode Island, USA; 3 Department of Medicine, Rhode Island Hospital, Brown University Health, Alpert Medical School of Brown University, Providence, Rhode Island, USA; 4 Departments of Neuroscience and Biology, Brown University, Providence, Rhode Island, USA

**Keywords:** Fetal Alcohol Spectrum Disorder, Temporal Lobe, Dietary Soy, Insulin Signaling, Notch, Behavior, Rat Model, Wnt, Gene Expression, GnRH, Prenatal Alcohol Exposure

## Abstract

**Background:**

Neurodevelopmental abnormalities in experimental fetal alcohol spectrum disorder (FASD) are associated with impaired signaling through complex pathways that mediate neuronal survival, growth, migration, energy metabolism, and plasticity. Gestational dietary soy prevents alcohol-related impairments in placentation and FASD-associated fetal anomalies.

**Objective:**

This study was designed to determine if gestational dietary soy would be sufficient to normalize cognitive function in young adolescent offspring after chronic in utero exposure to alcohol. In addition, efforts were made to characterize the mechanisms of FASD prevention by maternal dietary soy.

**Methods:**

Pregnant Long Evans rats were fed isocaloric liquid diets containing 0% or 26% caloric ethanol with casein or soy isolate as the protein source from gestation day 6 through delivery/postnatal day 0 (P0). From P24 - P28, the offspring were subjected to Morris water maze (MWM) testing, and on P35, they were sacrificed to harvest temporal lobes for histopathologic and molecular studies.

**Results:**

The in-utero ethanol-exposed offspring exhibited significant performance impairments on the MWM test, and they had a significantly reduced mean brain weight with neuronal loss in the CA1 hippocampal region and evidence of white matter myelin loss. Gestational dietary soy nearly normalized MWM performance and preserved brain weight, hippocampal CA1 architecture, and white matter myelin staining in alcohol-exposed offspring. Mechanistically, the main positive effects of soy included increased temporal lobe expression of HES-1 and HIF-1*α*, reflecting enhanced Notch signaling, and broadly increased expression of GnRH network molecules, including Erb1, Gper1, GnRH, GnRH-R, KiSS, and KiSS-R, irrespective of gestational ethanol exposure.

**Conclusions:**

Dietary soy intervention early in pregnancy may reduce FASD-associated cognitive deficits. The findings suggest that targeting Notch and GnRH-related networks may help reduce long-term disability with FASD. Additional mechanistic and experimental research is needed to determine if longer-duration, postnatal dietary soy could prevent the adverse neurobehavioral effects of FASD.

## 1. Introduction

Fetal Alcohol Spectrum Disorder (FASD) is caused by gestational ethanol exposure via maternal consumption during pregnancy [1] [2]. FASD is difficult to diagnose without attention to the constellation of characteristic but often subtle craniofacial features including a long, flat filtrum, low-set ears, a thin upper lip, epicanthal folds, ptosis, and midface hypoplasia [2]. FASD can also be associated with microcephaly and cognitive-motor deficits ranging from mild due to attention deficit hyperactivity disorders to severe with intellectual disability. Although not absolute, the severity of the phenotypic and neurobehavioral features of FASD is proportional to the burden of alcohol consumption in pregnancy [2].

Mechanistically, FASD-related abnormalities in the offspring are linked to impairments in placentation mediated in part by the inhibition of signaling through insulin and insulin-like growth factor (IGF) pathways [3]. Consequences include reduced expression and function of aspartyl-asparaginyl-*β*-hydroxylase (ASPH), which has critical roles in cell motility and adhesion [4]–[7] needed for placentation and fetal support [8]. ASPH is also functionally important for central nervous system development. The inhibition of ASPH expression has been linked to impaired neurodevelopment with disordered neuronal migration in experimental FASD [9] or molecular silencing [10]. Mechanistically, ASPH activates Notch by catalytic hydroxylation of Asp and Asn residues in its EGF-like domains [11] [12]. Activated Notch upregulates transcription of hairy and enhancer of split-1 (HES-1) and related molecules [11] [13] needed to regulate diverse developmental functions such as growth and maturation [14]. In addition, Wnt pathway signaling modulates neurodevelopment [15] and cross-talks with insulin/IGF [16] [17] and Notch [18] [19]. The inhibitory effects of ethanol on insulin/IGF, Notch, and Wnt pathway signaling in placenta and brain have been well documented [13]. Another pathway not thoroughly evaluated in relation to FASD or alcohol-related brain degeneration is the gonadotropin-releasing hormone (GnRH) network, which is also inter-related with insulin/IGF, Notch, and Wnt in adolescent and mature brains [20]. Moreover, growing evidence supports roles for GnRH networks in normal neurodevelopment such that dysregulation of GnRH signaling leads to cognitive impairment, and therapeutic rescue can be achieved by treatment with GnRH replacement analogues [21]–[23]. Therefore, it is of interest to examine the integrity of GnRH networks in relation to postnatal brain development in models of FASD.

Public health preventive measures are needed to more effectively consider the big picture related to maternal, placental, and fetal effects of prenatal alcohol exposures and the long-term consequences in the offspring [24]. Ideally, the approaches should take advantage of knowledge gained from basic and translational research. Given the importance of insulin/IGF, Notch, and Wnt networks during development and the considerable array of functional deficits that result from impairments in signal transduction and gene expression, efforts should be made to fortify the integrity of these pathways in the context of prenatal and perinatal alcohol exposures. Basic and translational research led to the concept that insulin sensitizers that also provide antioxidant and anti-inflammatory support could address problems stemming from prenatal alcohol exposures, including FASD. To this end, we evaluated the potential therapeutic effects of small molecule peroxisome-proliferator-activated receptor (PPAR) agonists, dietary soy, and bioactive soy constituents in experimental models rendered insulin resistant due to alcohol exposure, high fat diet-induced obesity, or nitrosamine administration, and demonstrated promising therapeutic effects marked by reversal or prevention of neurodevelopmental pathologies [25]–[30]. Successes with the use of PPAR agonists were attributed to the ability of these small molecules to bypass plasma membrane surface receptors and directly modulate gene transcription in the nucleus [31] [32]. Unfortunately, PPAR agonist drugs cannot be broadly offered to pregnant women due to unpredictable harm across the maternal-placental-fetal axis. Instead, a more practical approach would be to take advantage of the beneficial effects of natural insulin-sensitizing substances such as soy or its bioactive isoflavones, daidzein, and genistein [33]. Correspondingly, in recent studies, we showed that chronic feeding with dietary soy minimized or prevented the adverse effects of prenatal alcohol exposures on placentation and fetal development [3], and that later adolescent exposures positively impacted cognitive-behavioral function [34]–[36]. However, our follow-up question was whether dietary soy administered only in pregnancy would suffice to prevent FASD-related neurodevelopmental pathologies that emerged early in adolescence. The present study examines the effects of discontinuing chronic alcohol exposures and dietary soy feeding at birth. Postnatal brain development, neurobehavioral function, and insulin/IGF, Notch, Wnt, and GnRH pathway signaling molecule expression were evaluated to characterize potential mediators of recovery or persistence of FASD.

## 2. Materials and Methods

### Materials:

The bicinchoninic acid (BCA) kit to measure protein concentration was purchased from Pierce Chemical Co (Rockford, IL). Histochoice fixative was purchased from Amresco, Inc (Solon, OH). Amplex UltraRed soluble fluorophore, and the Akt Pathway Total and Phospho 7-Plex panels were purchased from Invitrogen (Carlsbad, CA). Maxisorp 96-well plates used for ELISAs were from Nunc (Thermo Fisher Scientific; Rochester, NY). Superblock-TBS, horseradish peroxidase conjugated antibodies were from Pierce Chemical Co (Rockford, IL). All other monoclonal antibodies and immunodetection reagents used for enzyme-linked immunosorbent assays (ELISAs) were purchased from Abcam (Cambridge, MA), Upstate (Billerica, MA), Vector Laboratories (Burlingame, CA), Invitrogen (Carlsbad, CA) or Chemicon (Temecula, CA). The AMV 1st Strand cDNA Synthesis Kit was purchased from Roche Applied Science (Indianapolis, IN). Synthetic oligonucleotides used in quantitative polymerase chain reaction (qPCR) assays were purchased from Sigma-Aldrich Co (St. Louis, MO). QIAzol Lysis Reagent for RNA extraction, QuantiTect SYBR Green PCR Mix, and the BIO Robot Z1 were from Qiagen, Inc (Valencia, CA). Fine chemicals were purchased from CalBiochem (Carlsbad, CA), or Sigma-Aldrich (St Louis, Mo).

### Experimental model:

The study was conducted according to the guidelines of the Declaration of Helsinki and approved by the Institutional Animal Care and Use Committee (IACUC) at Rhode Island Hospital and Lifespan (Committee #503823 approved 09/28/2023), and the protocol adheres to the National Institutes of Health (NIH) Guide for the Care and Use of Laboratory Animals. To generate the experimental model, timed-pregnant Long Evans rats from Charles River Laboratories (Willmington, MA, USA) were fed with isocaloric liquid diets (BioServ, Frenchtown, NJ) that contained 0% (control) or 26% pharmaceutical grade ethanol (caloric content) with casein (standard, control) or soy isolate as the protein source [3]. The resulting four experimental groups were designated as: 1) CC for control-casein; 2) CS, control-soy; 3) EC for ethanol-casein; and 4) ES for ethanol-soy. The liquid diets were initiated on gestation day 6 to minimize the adverse effects of alcohol exposure on implantation and discontinued on postnatal day 0 (P0; delivery), after which all diets were switched to standard chow. Pregnant dams were housed in pairs. Dams with litters were housed in separate cages.

The pups were weighed at birth and then weekly and monitored daily. The weaned offspring (CC, n = 9; EC, N = 13; CS, n = 10; EC, n = 15) were housed in same-sex cages in a pathogen-free animal facility with an automated 12-hour light/dark cycle (Lights on at 7:00 AM and Lights off at 7:00 PM) and maintained on chow ad libitum with free access to water. Enrichment toys were not used due to possible differential effects on neurobehavioral performance in alcohol exposure models [37]. Morris Water Maze (MWM) testing was performed over 4 consecutive days with 3 daily trials from P25-P28. On P35 (5 Weeks), the rats were sacrificed using isoflurane. After harvesting the brains, one temporal lobe was immersion fixed in 10% neutral buffered formalin and processed by paraffin embeding, and the other was snap-frozen on dry ice and stored at −80°C for molecular and biochemical studies. Formalin-fixed paraffin-embedded histological sections (5 μm thick) were stained with Luxol fast blue/hematoxylin and eosin (LHE) to reveal neuronal and glial cytomorphology and white matter fibers.

### Morris Water Maze:

Morris Water Maze (MWM) testing assessed spatial learning and memory [38]. In brief, rats were subjected to 3 daily trials in which the latencies (seconds) required to locate and land on the platform were recorded. On Day 1, the platform was visible, but on Days 2 – 4, the platform was submerged. On Days 3 and 4, the maze entry quadrants were randomized for each trial. The rats were allowed 120 sec to locate the platform, after which they were guided. Recordings of the trials were analyzed using the Ethovision 13.0 software. Area-under-curve calculations were made for the 3 daily trials. Data were analyzed by ANOVA with post hoc multiple comparison tests.

### Multiplex Enzyme-Linked Immunosorbent Assay (ELISA):

For molecular studies of immunoreactivity and mRNA, 6 randomly selected samples from each group were analyzed using multiplex and duplex ELISAs. The sample sizes were based on power analysis calculations using preliminary and prior data. For the multiplex ELISAs, temporal lobe tissue samples (100 mg) were homogenized in 5 volumes of Weak Lysis Buffer (150 mM NaCl, 50 mM Tris-Base pH 7.5, 0.1% Triton X-100, 5 mM EDTA pH 8.0, 10 mM EDTA, 50 mM NaF) that contained protease inhibitor cocktail and phosphatase inhibitors as described above [39]. For the duplex ELISAs, tissue homogenates were prepared in five volumes of radioimmunoprecipitation assay (RIPA) buffer containing protease and phosphatase inhibitors. Protein content was determined using the Bicinchoninic (BCA) Assay.

Magnetic multiplex bead-based Luminex platform kits (Invitrogen, Carlsbad, CA USA) measured total and phosphorylated proteins in the insulin/IGF-1/IRS-Akt pathway, downstream through Akt including: insulin receptor (IR), tyrosine phosphorylated IR (pYpY1162/1163-IR), IGF-1 receptor (IGF-1R), tyrosine phosphorylated IGF-1R (pYpY1135/1136-IGF-1R), IRS-1, serine-phosphorylated IRS-1 (pS312-IRS-1), Akt, pS473-Akt, glycogen synthase kinase 3*β* (GSK-3*β*), pS9-GSK3*β*, proline-rich Akt substrate of 40 kDa (PRAS40), pT246-PRAS40, 70-kDa ribosomal protein S6 kinase (p70S6K), and pTpS421/424-p70S6K following the manufacturer’s protocol [40]. Temporal lobe homogenates containing 100 μg protein were incubated with the beads, and captured antigens were detected with biotinylated secondary antibodies and phycoerythrin-conjugated Streptavidin. Results were measured in a MAGPIX with xPONENT software. Data are expressed in arbitrary fluorescence light units (FLU) based on standard curves.

Duplex ELISAs measured immunoreactivity to ASPH using the A85G6 and A85E6 monoclonal antibodies [9], Notch 1, Jagged1, hairy/enhancer of split-1 (HES1), hypoxia-inducible factor-1 (HIF-1*α*), and factor inhibiting HIF-1 (FIH). Temporal lobe protein homogenates (40 ng/100μl) adsorbed to the bottom surfaces [41] of 96-well Maxisorb plates [42] overnight at 4°C were blocked for 3 hours with 3% BSA in Tris buffered saline (TBS), then incubated with primary antibody (0.1 – 1.0 μg/ml) for 1 hour at room temperature. Immunoreactivity was detected with horseradish peroxidase (HRP)-conjugated secondary antibody (1:10000) and Amplex Ultra Red soluble fluorophore [42]. Amplex Red fluorescence was measured (Ex 579/Em 595) in a SpectraMax M5 microplate reader (Molecular Devices Corp., Sunnyvale, CA). Negative control reactions were performed with antibody omissions. Immunoreactivity was normalized to large acidic ribonuclear protein (RPLPO) measured in the same samples as a loading control [43].

### Duplex Quantitative Reverse Transcriptase Polymerase Chain Reaction (qRT-PCR) Analysis:

Quantitative reverse-transcriptase, polymerase chain reaction (qRT-PCR) analysis was used to measure Notch pathway mRNA transcripts, including ASPH, Notch 1, Jagged1, HES1, HIF-1*α*, and FIH, and selected Wnt canonical and non-canonical pathway mRNA transcripts, including Wnt5a, Wnt5b, Frizzled 4 (Fzd4), Fzd6, E1a Binding Protein 300 (EP300), Dixdc1, and Axin2 mRNA. RNA extracted from fresh-frozen tissue using the RNeasy Mini Kit (Qiagen, Valencia, CA, USA) was reverse-transcribed with random oligonucleotide primers and the AMV 1st Strand cDNA Synthesis Kit. Gene expression was measured using a hydrolysis probe-based duplex qRT-PCR assay in which *β*-actin served as a reference gene [11] [44]. Reactions (20 μL) contained Taqman Gene expression master mix, 200 nM of gene-specific and *β*-actin primers, 100 nM of *β*-actin (Y555-labeled), and gene of interest (FAM-labeled) probes. The Probe-Finder Software (Roche, Indianapolis, IN) determined gene-specific primer sequences and matched probes. PCR amplifications were initiated by a 10-minute, 95°C denaturation step and followed by 45 2-step cycles of denaturation (15 seconds at 95°C) and annealing/extension (1 minute at 60°C). The PCR amplifications were performed in a LightCycler 480 PCR machine (Roche, Indianapolis, IN). Fluorescence signals corresponding to the genes of interest were acquired in the FAM Channel (Em: 483 – 533 nm), and the *β*-actin signal was acquired in the Y555/HEX channel (Em: 523 – 568 nm). Results were analyzed using LightCycler Software 4.0.

### PCR Array Studies of the GnRH Pathway:

A custom targeted PCR array was generated to measure mRNA levels of Estrogen Receptor 1 (Esr1), Estrogen Receptor b1 (Erb1), G-Protein Coupled Estrogen Receptor (Gper), Gonadotropin releasing hormone (GnRH), GnRH-receptor (GnRH-R), Kisspeptin 1 (KiSS1), KiSS1R), hypoxanthine phosphoribosyltransferase (HPRT1), and *β*-Actin (See Table 1). The goal was to examine the effects of prenatal ethanol and dietary soy exposures on genes relevant to CNS neuronal plasticity during adolescent development [22] [41] [45]–[47] but not previously analyzed in relation to FASD. PCR primer pairs were designed with Primer 3 (http://www.ncbi.nm.gov/tools/primer-blast/) software (Table 1). Targeted arrays were constructed by spotting and drying primer pairs (100 nmol/5 μl) into individual wells of a Lightcycler 480 Multi-well Plate 96 (Roche, Indianapolis, IN). Reaction cocktails (20 μl) containing cDNA from 5 μg RNA template and Sybr green master mix were added to each well. PCR reactions were performed in a Roche Lightcycler 480 System and gene expression was analyzed using the ^ΔΔ^C_t_ method with results normalized to the HPRT1 and *β*-Actin control genes.

### Statistical Analysis:

Neurobehavioral testing, image analysis, and molecular data acquisition were performed under code. Statistical analysis was performed, and graphs were generated using the GraphPad Prism 10.2 software (GraphPad Software, Inc., San Diego, CA.). Inter-group comparisons were made by analysis of variance (ANOVA) with post hoc Tukey tests of significance. Statistical significance was set at p ≤ 0.05. Statistical trends defined as 0.05 < p < 0.10 were also noted. Intergroup comparisons were made by analysis of variance (ANOVA) with post hoc multiple comparison tests.

## 3. Results

### Effects of ethanol on body and brain weight:

The pregnant dams in all groups progressively gained weight such that at delivery (GD21), there were no significant inter-group differences in body weight (Table 2). On P0, there were no significant differences in the mean birth weights among the four offspring groups, but by P35, the experimental endpoint, inter-group differences were detected (p = 0.0006) due to the significantly higher ES mean body relative to the other three groups. Regarding brain weight at P35, significant inter-group differences (p < 0.0001) were detected by ANOVA due to the EC-associated reduction relative to the other three groups (Table 2). In contrast, the ES group had a normalized mean brain weight relative to CC and CS rats, reflecting a rescue/preventive effect of gestational dietary soy.

### Neurobehavioral Testing:

**Figure 1.**
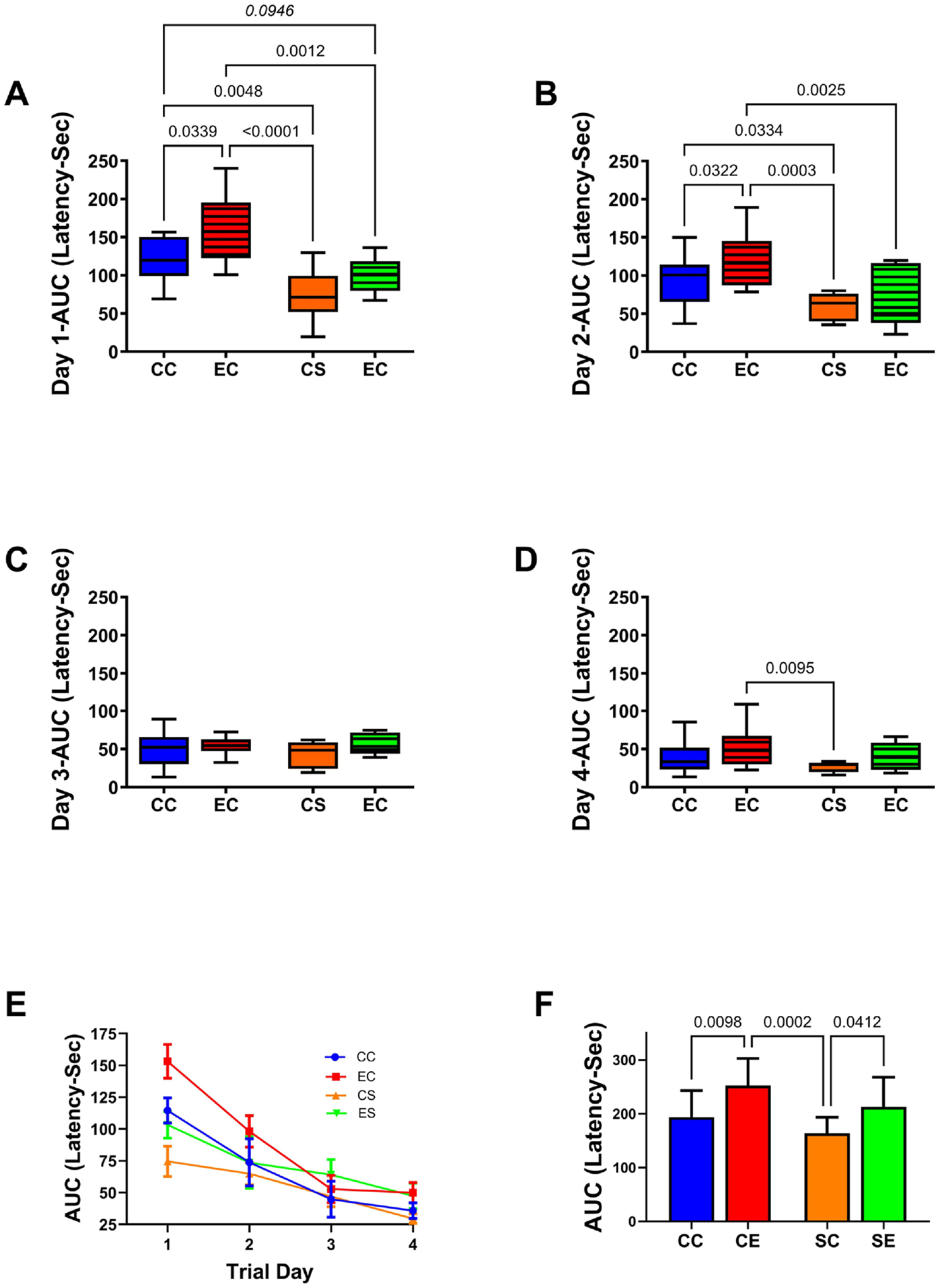
Effects of ethanol and dietary soy on spatial learning and memory-morris water maze (MWM) test. MWM testing was performed on four consecutive days from postnatal day (P) 25 through P28. The latencies required to reach and land on the platforms were tracked and scored with Ethovision software. Area-under-curve calculations over the 3 daily trials were used for inter-group comparisons. Box plots show performance differences on (A) Trial Day 1, (B) Trial Day 2, (C) Trial Day 3, and (D) Trial Day 4. (E) Linear trajectories of performance over the 4 trial days. Data points reflect the mean + S.D. of the AUC latencies. (F) Bar graphs depict the mean ± S.D. of the AUCs corresponding to the curves in Panel E. The data were analyzed by ANOVA. Significant (p ≤ 0.05) post hoc intergroup differences and statistical trend wise differences (italics) are displayed in the panels.

The MWM test assessed spatial learning and memory. MWM testing was conducted over 4 consecutive days with 3 trials per day. Data were analyzed by calculating the AUC for performance (latency to arrive and land on the platform) over the three daily trials. Longer latency reflects worse performance. The Kruskal-Wallis test demonstrated significant intergroup differences in MWM performance on Trial Day 1 (KW = 21.33; p < 0.0001) and Day 2 (KW = 16.21, p = 0.001), and a statistical trend effect on Trial Day 4 (KW = 6.925, p = 0.074). Post hoc tests demonstrated significantly longer mean latencies to locate and land on the platform, *i.e*., worse performance in the EC relative to the CC, CS, and ES groups on Trial Days 1 and 2 (Figure 1(A) and Figure 1(B)). In addition, on Trial Days 1 and 2, the mean latencies in the CS group were significantly shorter than in the CC control group, and the ES group exhibited a trend decrease in latency relative to CC on Trial Day 1. On Trial Day 3, there were no significant intergroup differences in performance (Figure 1(C)). On Trial Day 4 (Figure 1(D)), the CS group exhibited a significantly shorter mean latency relative to EC. Also, in contrast to the performance deficits observed in EC versus CC, no significant differences between CS and ES were detected throughout the study. To further address this point, line graphs were generated to compare performance trends over time (Figure 1(E)). The curves demonstrated the most prominent inter-group separations on Trial Day 1 followed by Trial Day 2, and substantial overlap on Trial Days 3 and 4. However, the AUCs calculated for the longitudinal trends demonstrated a significantly longer mean latency in EC relative to CC and SC, and in ES relative to CS (Figure 1(F)) indicating that the EC group exhibited the worst overall performance, and although the ES group’s performance was relatively normalized, it was not as good as in the CS group.

### Temporal Lobe/Hippocampal Pathology:

**Figure 2.**
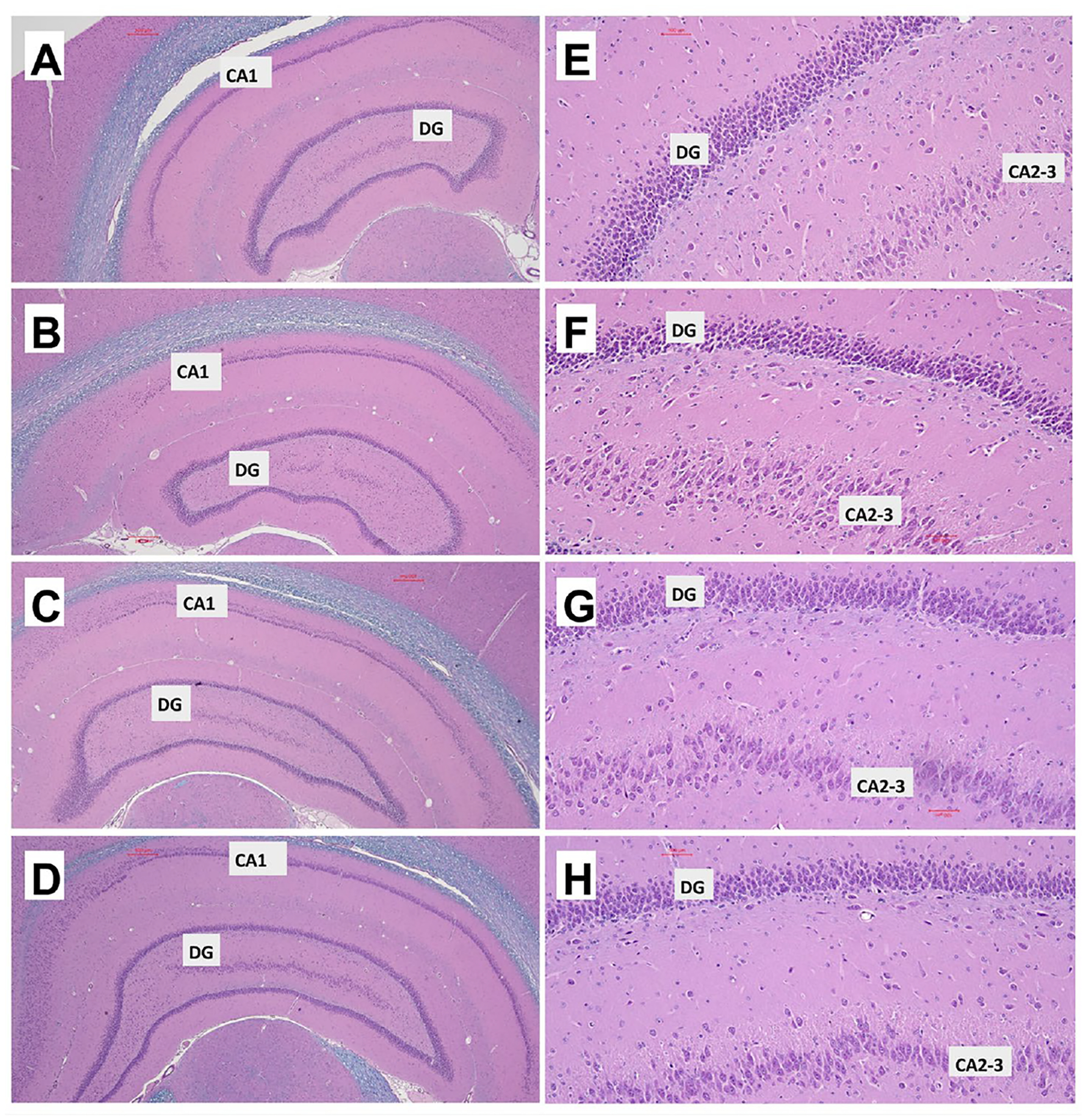
Hippocampal structural changes following gestational ethanol and dietary soy exposures via pregnant dams. Formalin-fixed, paraffin-embedded histological sections (5 μm thick) of hippocampal formation including (CA1 and dentate gyrus (DG) regions with adjacent white matter (wm) and temporal cortex (TC) from (A), (E) CC, (B), (F) EC, (C), (G) CS, or (D), (H) ES P35 rats were stained with Luxol Fast Blue-Hematoxylin and Eosin (LHE). Note the overall more elongated shapes and tapered ends of the hippocampal formations in the CS and ES (C), (D) relative to CC and EC (A), (B) samples. (E)-(H) Higher magnification images that include DG and CA2–3 show subtle possible reductions in the neuronal cell densities in (F) EC relative to (E) CC. However, the overall hippocampal structures are relatively preserved despite gestational ethanol exposures. Original magnifications: (A)-(D) = 20x; (E)-(H) = 400x; Scale bars = 100 μm.

**Figure 3.**
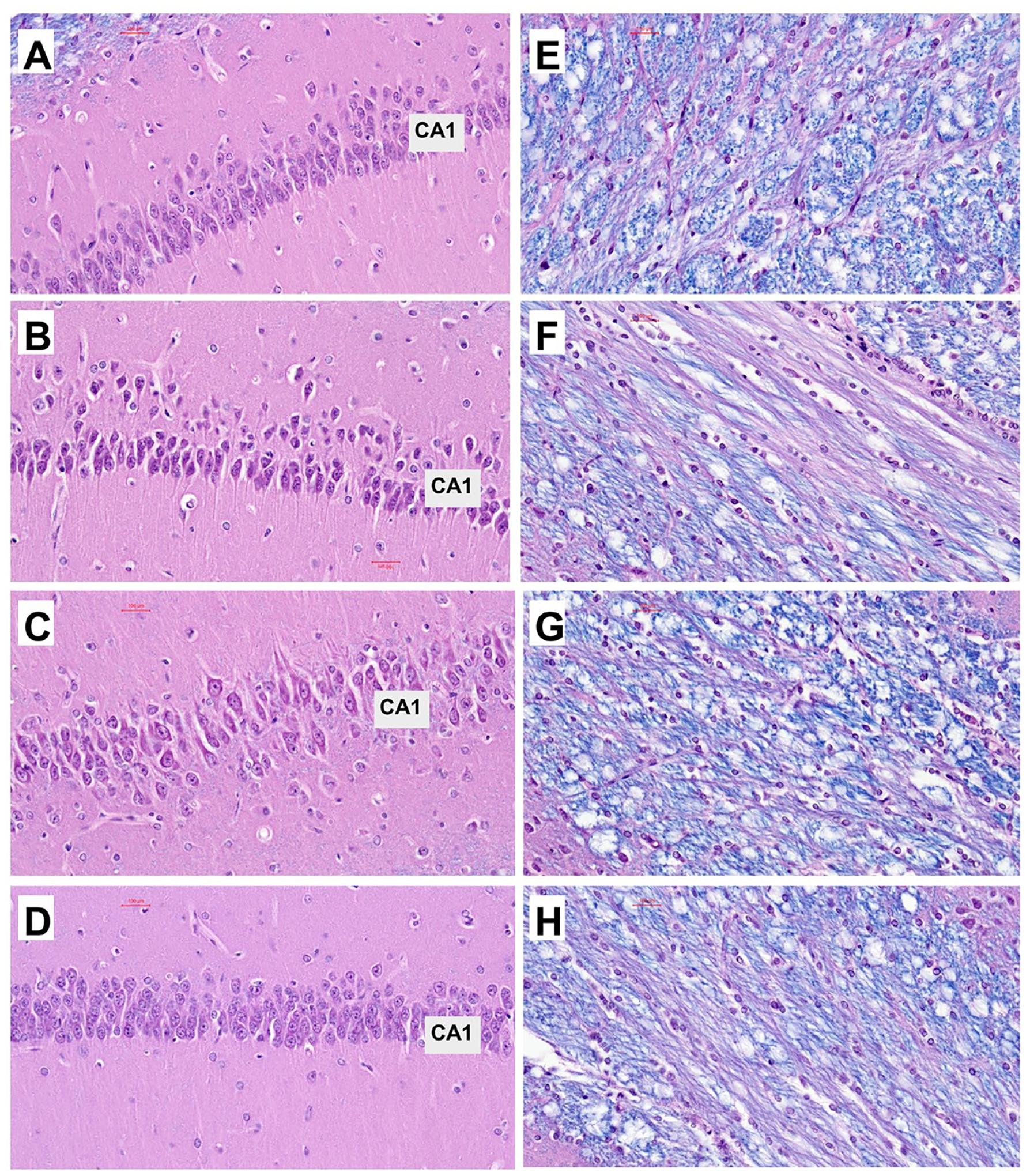
Ethanol and dietary soy effects on the CA1 and white matter tract regions of the hippocampal formation. (A)-(D) CA1 region and (E)-(H) adjacent white matter fibers (see Figure 2) in representative hippocampi from (A), (E) CC, (B), (F) EC, (C), (G) CS, and (D), (H) ES P35 brains. Formalin-fixed, paraffin-embedded histological sections (5 μm thick) were stained with LHE. Note lower density and less compact neuronal architecture in EC compared with CC, CS, and ES, and pronounced pallor of the white matter fibers (arrows) in EC compared with the other 3 groups. In ES (H), the Luxol fast blue myelin staining pallor is considerably less than in EC and nearly normalized relative to CC (E) and CS (G). Original magnifications: (A)-(D) = 20x; (E)-(H) = 400x; Scale bars = 100 μm.

Histological sections of the temporal lobe with hippocampal formation and adjacent white matter tracks and overlying cortex were stained with hematoxylin and eosin(H&E) and luxol fast blue (LFB). The goal was to compare the effects of chronic gestational ethanol exposure and maternal dietary soy on hippocampal architecture (H&E) and white matter myelin integrity (LFB). Low-magnification images revealed well-developed hippocampi with similar overall architectural features in all four groups (Figure 2). Only subtle differences manifested by hippocampal formation with more prominent elongation and tapered medial and lateral ends in the CS (Figure 2(C)) and ES (Figure 2(D)) compared with CC (Figure 2(A)), and EC (Figure 2(B)) samples. Higher magnification images of the dentate gyrus and CA2–3 region of Ammon’s horn revealed subtle differences marked by tighter laminar architectures which may reflect higher neuronal densities in the CS (Figure 2(G)) and ES (Figure 2(H)) versus CC (Figure 2(E)) and EC (Figure 2(F)) samples. In contrast, the effects of ethanol and dietary soy were more apparent in the CA1 hippocampal region (Figures 3(A)–(D)) and white matter fibers (Figures 3(E)–(H)). In contrast to the compact cellular layer with uniform populations of neurons in the CC CA1 hippocampal region (Figure 3(A)), EC samples exhibited reduced neuronal populations with many shrunken pyknotic (dense dark) nuclei (Figure 3(B)). The CS CA1 region (Figure 3(C)) was well-populated by pyramidal neurons that appeared somewhat larger with sharper delineations than in CC. The ES CA1 region was well populated by uniform and compactly positioned neurons (Figure 3(D)), similar to the features in CC. In CC (Figure 3(E)) and SC (Figure 3(G)) white matter, the LFB staining of myelinated fibers was associated with delicately branched fibrovascular elements stained with H&E. In contrast, EC resulted in a conspicuous reduction in LFB myelin staining and less conspicuous fibrovascular elements (Figure 3(F)). Dietary soy feeding of the dam resulted in relatively modest reductions in white matter myelin LFB staining and preservation of the fibrovascular network (Figure 3(H)).

### Multiplex Immunoassays of Insulin Receptor/IGF-1 Receptor/IRS-1 Pathway:

Previous studies showed that neurobehavioral dysfunction mediated by chronic ethanol exposures impair signaling through insulin and IGF-1 receptors, IRS-1, and Akt while increasing GSK-3*β* activation in the CNS [4] [42] [48]–[52]. In addition, we found that dietary soy can reverse or prevent neurobehavioral, phenotypic features, and CNS molecular pathologies in an adolescent alcohol exposure model [34]. Herein, we examined the developmental effects of discontinuing ethanol and dietary soy exposures at birth, contrasting with previous studies in which the analyses were performed in models with chronic plus binge ethanol administrations [50]. To examine the extent to which dietary soy’s therapeutic or rescue effects in the FASD model were mediated by restoration of insulin/IGF-1/IRS-1/Akt pathway signaling, the temporal lobe tissue samples were analyzed with multiplex total and phosphoprotein ELISAs. In addition, the calculated relative levels of phosphorylation (pY, pS or pT-/total protein) were compared. Two-way ANOVA detected significant dietary protein source effects on the expression of the IGF-1R, IRS-1, PRAS40, pY-IGF-1R, pT-PRAS40, pTpS-P70S6K, pY-IGF-1/total IGF-1, and pT-PRAS40/total PRAS40 (Table 3). The only significant ethanol effects were associated with pT-PRAS40 and pT-PRAS40/total PRAS40. Statistical trend effects were observed for dietary protein effects on pS-Akt, ethanol effects on pS-GSK-3*β*, and diet × protein interactions regarding pT-PRAS40/total PRAS40.

**Figure 4.**
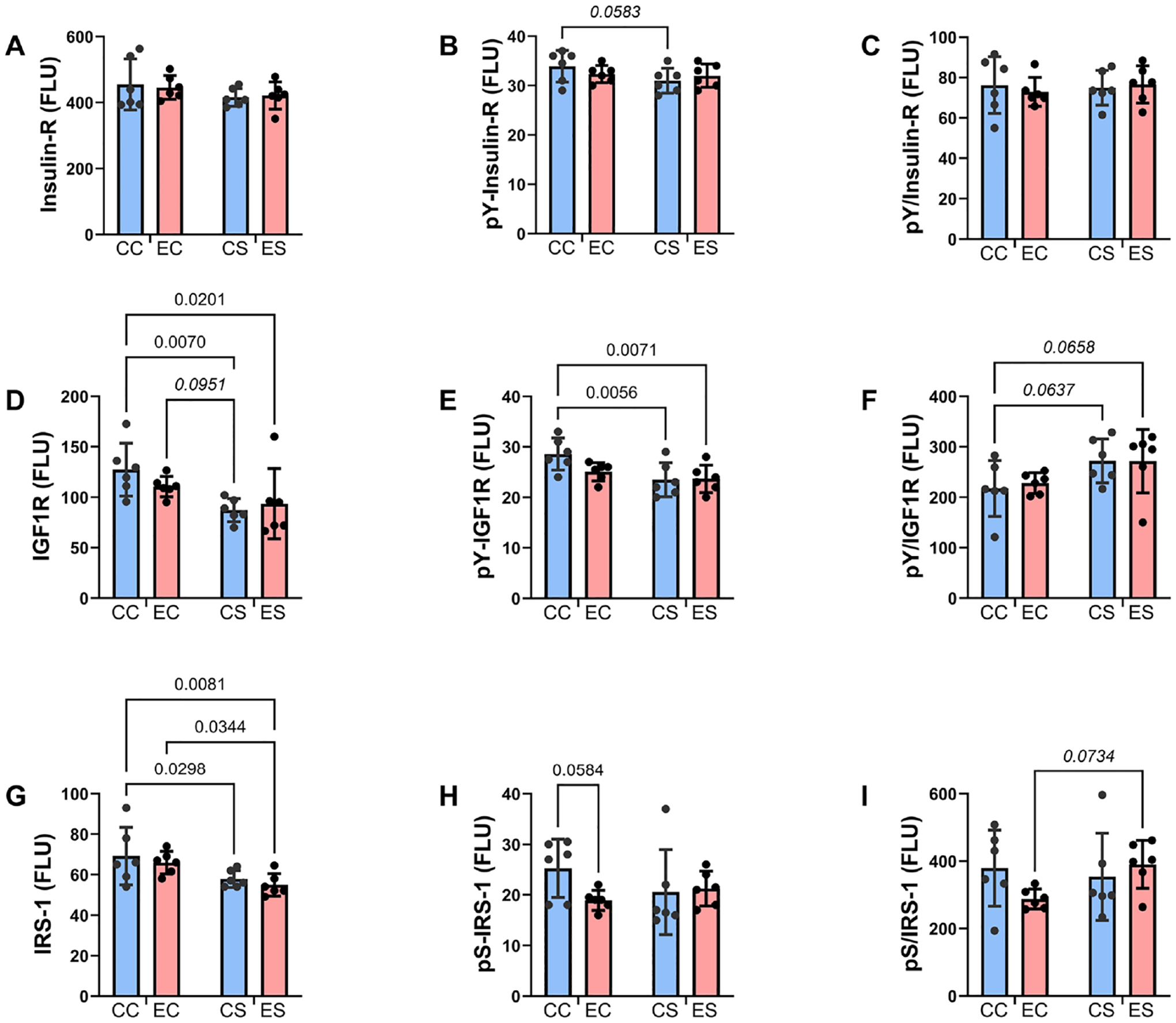
Upstream insulin and insulin-like growth factor signaling molecules. Homogenates of the temporal lobe with hippocampal formation were used to measure (A) insulin receptor (Insulin-R), (D) IGF-1R, (G) IRS-1, (B) ^pYpY1162/1163-^Insulin-R, (E) ^pYpY1135/1136-^IGF-1R, and (H) ^pS312-^IRS-1 with Total and Phospho Akt 7-Plex Panels. Relative levels of phosphorylation are represented by the calculated ratios of (C) ^pYpY1162/1163-^/Insulin-R, (F) ^pYpY1135/1136-^/IGF-1R, and (I) ^pS312^/IRS-1. N = 8 in each of the CC, EC, CS, and ES groups. See the two-way ANOVA test results in Table 3. Significant (p ≤ 0.05) and statistical trend wise (0.05 < p < 0.10; italics) differences detected with post hoc tests are displayed within the panels.

**Figure 5.**
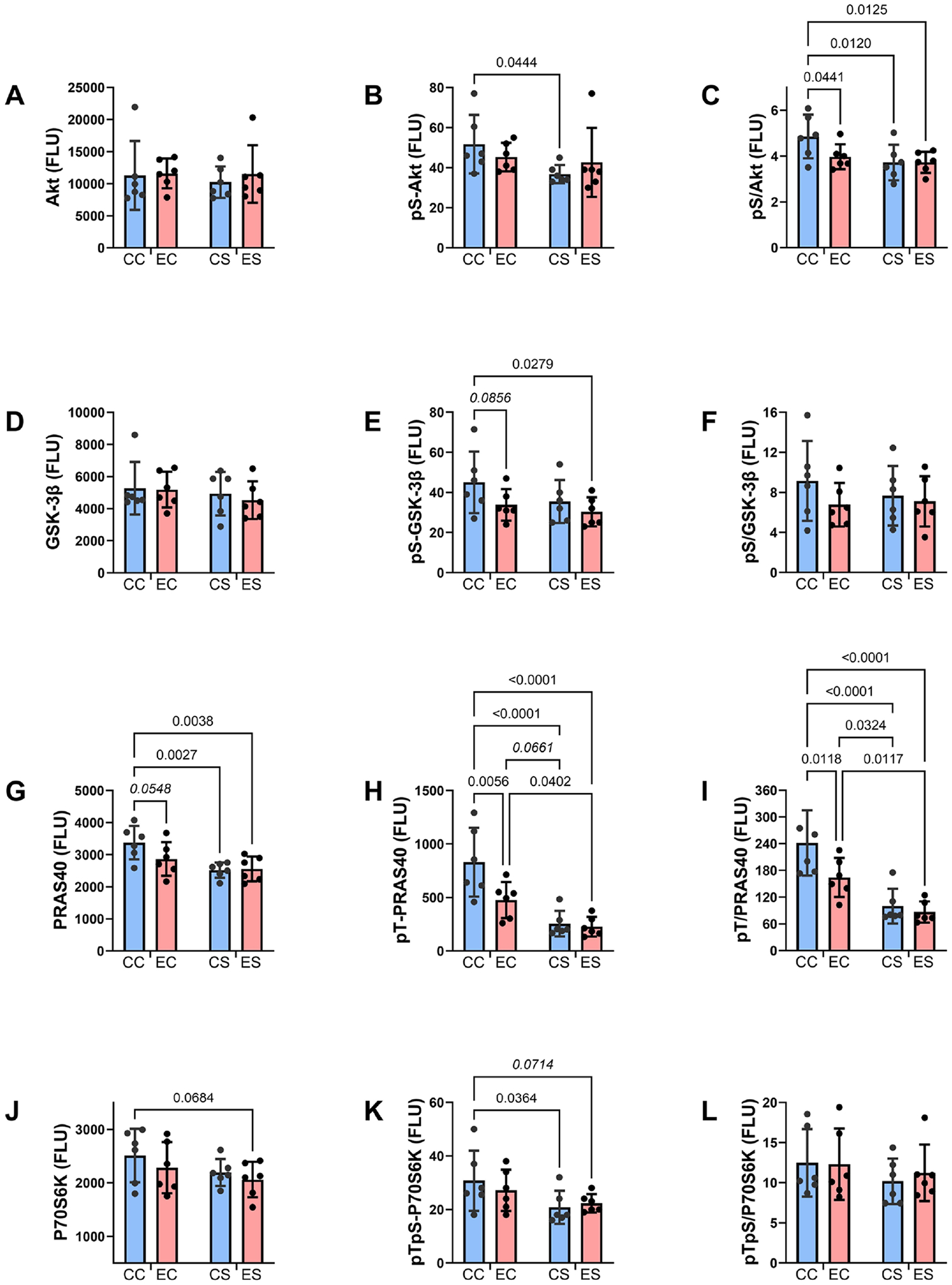
Effects of chronic gestational exposures to ethanol and dietary soy on downstream insulin and IGF pathway signaling. Homogenates of the temporal lobe were used to measure (A) Akt, (D) GSK-3*β*, (G) PRAS40, (J) P70S6K, (B) ^pS473^-Akt, (E) ^pS9^-GSK-3*β*, (H) ^pT246^-PRAS40, and (K) ^pTpS421/424^-P70S6K, with the Total and Phospho Akt 7-Plex Panels. Relative degrees of phosphorylation are shown by the calculated ratios of (C) ^pS473-^/Akt, (F) ^pS9-^/GSK-3*β*, (I) ^pT240-^/PRAS40, and (L) ^pTpS421/424-^/P70S6K. N = 8 in each of the CC, EC, CS, and ES groups. See Table 3 for the two-way ANOVA test results. Significant (p ≤ 0.05) inter-group differences detected with post hoc tests are displayed within the panels.

Bar graphs display the effects of ethanol and dietary soy on insulin/IGF-1/IRS-1-Akt pathway proteins and phosphoproteins (Figure 4 and Figure 5). Regarding the upstream components of the pathway, the sources of significant variance by two-way ANOVA tests were due to similarly reduced levels of IGF-1R (Figure 4(D)) and pY-IGF-1R (Figure 4(E)) and trend-wise elevated pY-IGF-1R/total IGF-1R (Figure 4(F)). IRS-1 immunoreactivity was also reduced in CS and ES relative to EC and/or CC (Figure 4(G)). Otherwise, statistical trend-wise reductions in pY-Insulin-R in CS versus CC (Figure 4(B)) and pS-IRS-1 in EC relative to CC (Figure 4(H)), and an increase in pS-IRS-1/total IRS-1 in ES versus EC (Figure 4(I)) were observed. Note that since pS-IRS-1 is inhibitory to IRS-1 signaling [53], gestational dietary soy reduced the relative levels of IRS-1 signaling in ES versus EC. No significant or trend-wise effects were detected with respect to the Insulin-R (Figure 4(A)) or pY-Insulin-R/Total Insulin-R (Figure 4(C)).

Regarding the downstream signaling molecules, pS-Akt was significantly lower in CS than CC (Figure 5(B)), and pS-Akt/total Akt was significantly reduced in EC, CS, and ES relative to CC (Figure 5(C)). The mean levels of pS-GSK-3*β* were trend-wise or significantly reduced in EC and ES relative to CC (Figure 5(E)), reflecting higher levels of GSK-3*β* activation. Regarding PRAS40, the effects of ethanol and/or dietary soy were largely inhibitory relative to CC. PRAS40 expression was trend-wise reduced in EC and significantly reduced in CS and ES relative to CC (Figure 5(G)), and both pT-PRAS40 and pT-PRAS40/total PRAS40 were significantly reduced in EC, CS, and ES relative to CC (Figure 5(H) and Figure 5(I)). The mean levels of P70S6K (Figure 5(J)) and pTpS-P70S6K (Figure 5(K)) were trend-wise reduced in ES relative to CC, and pTpS-P70S6K was significantly reduced in CS relative to CC (Figure 5(K)). There were no significant or trend-wise ethanol or dietary soy effects on Akt (Figure 5(A)), GSK-3*β* (Figure 5(D)), pS-GSK-3*β*/total GSK-3*β* (Figure 5(F)), or pTpS-P70S6K/total P70S6K (Figure 5(L)).

### Notch Pathway Studies:

Notch pathway responses to ethanol and dietary soy were investigated at the mRNA and protein levels. Previous studies linked the adverse effects of prenatal alcohol exposure on neurodevelopment to impairments in signaling through Notch, including reduced expression of ASPH [13] [34] [44] which catalytically activates Notch via hydroxylation of a critical His-residue in EGF-like domains [54]. Using duplex RT-PCR assays with actin as the loading control, the relative levels of ASPH, Notch1, Jagged1, HES-1, HIF-1*α*, and FIH mRNA transcripts were measured in temporal lobe tissue. Two-way ANOVA tests detected significant dietary protein effects on HES-1 and HIF-1*α* and dietary protein × ethanol interactive effects on Jagged 1 mRNA levels (Table 4). The post hoc tests demonstrated higher levels of HES-1 in ES versus EC, and higher levels of HIF-1*α* in CS and ES compared with CC and EC (Supplementary Figure 1). Otherwise, there were no significant or trend-wise inter-group differences in ASPH, Notch1, Jagged1 or FIH mRNA levels.

**Figure 6.**
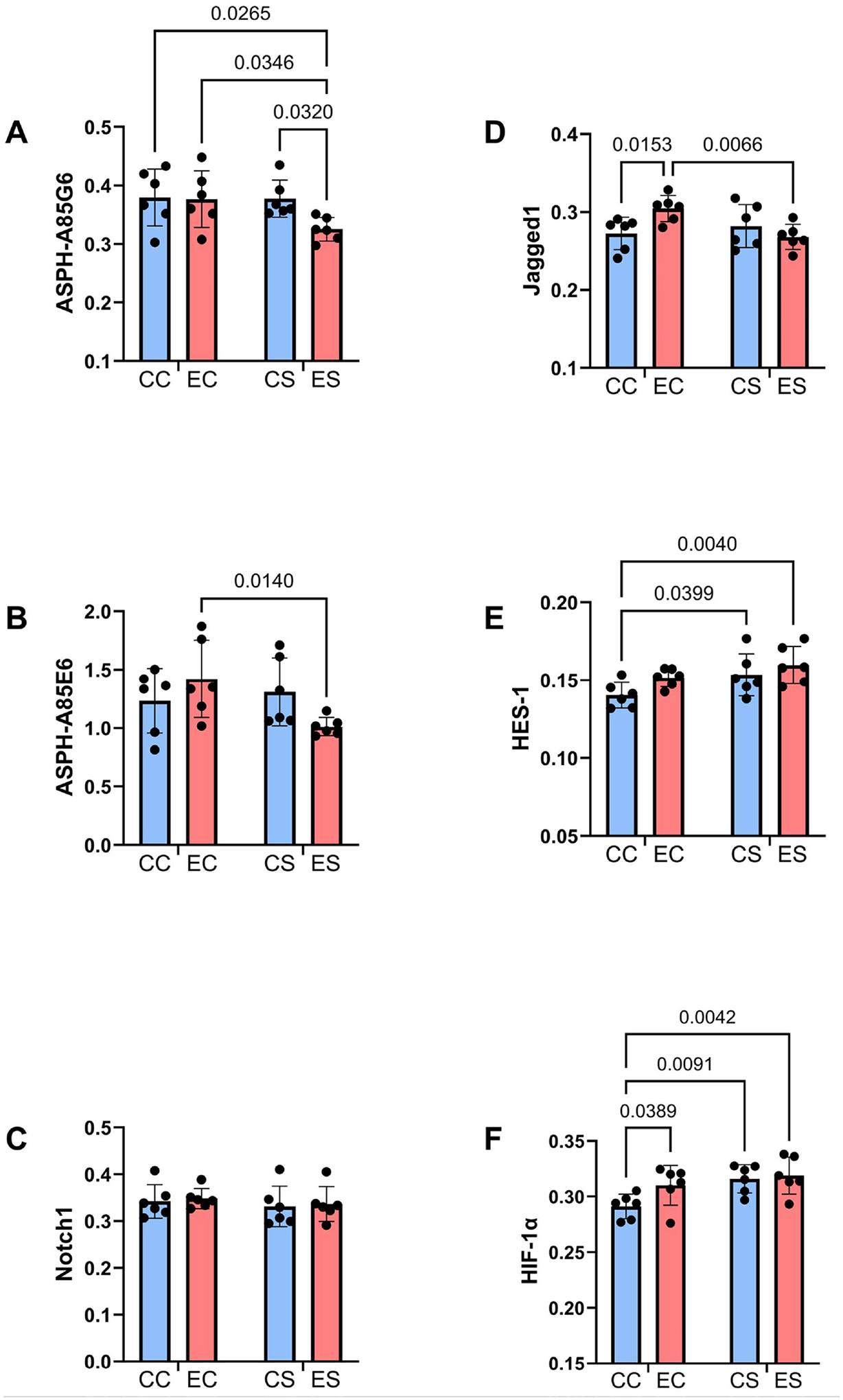
Ethanol and dietary soy effects on ASPH and notch networks. Immunoreactivity was measured by duplex ELISA with results normalized to RPLPO measured in the same samples. Graphs depict relative levels of immunoreactivity (mean ± S.D.) of (A) ASPH-A85G6, (B) ASPH-A85E6, (C) Notch1, (D) Jagged1, (E) HES1, and (F) HIF-1*α*. Two-way ANOVA tests were used for inter-group statistical comparisons (Table 5). The significant (p ≤ 0.05) multiple comparisons post-hoc Tukey test results are displayed within the panels.

Corresponding with the mRNA studies, two-way ANOVA tests demonstrated significant effects of dietary soy on HES-1 and HIF-1*α* immunoreactivity, and a dietary protein × ethanol interactive effect on Jagged1 (Table 5). In addition, a significant ethanol effect was observed for HES-1, and trend-wise effects of ethanol were observed for ASPH-A85G6 and HIF-1*α*. Graphs of the duplex ELISA results demonstrated significantly reduced mean levels of ASPH-A85G6 in ES relative to the other three groups (Figure 6(A)), and ASPH-A85E6 in ES relative to EC (Figure 6(B)). There were no significant inter-group differences in Notch1 immunoreactivity (Figure 6(C)). Jagged1 immunoreactivity was significantly higher in EC relative to both CC and ES (Figure 6(D)). The mean levels of both HES-1 (Figure 6(E)), HIF-1*α* (Figure 6(F)) were significantly higher in CS and ES compared to CC and HIF-1*α* was also elevated in EC relative to CC (Figure 6(F)).

### Wnt Pathway mRNA Studies:

**Figure 7.**
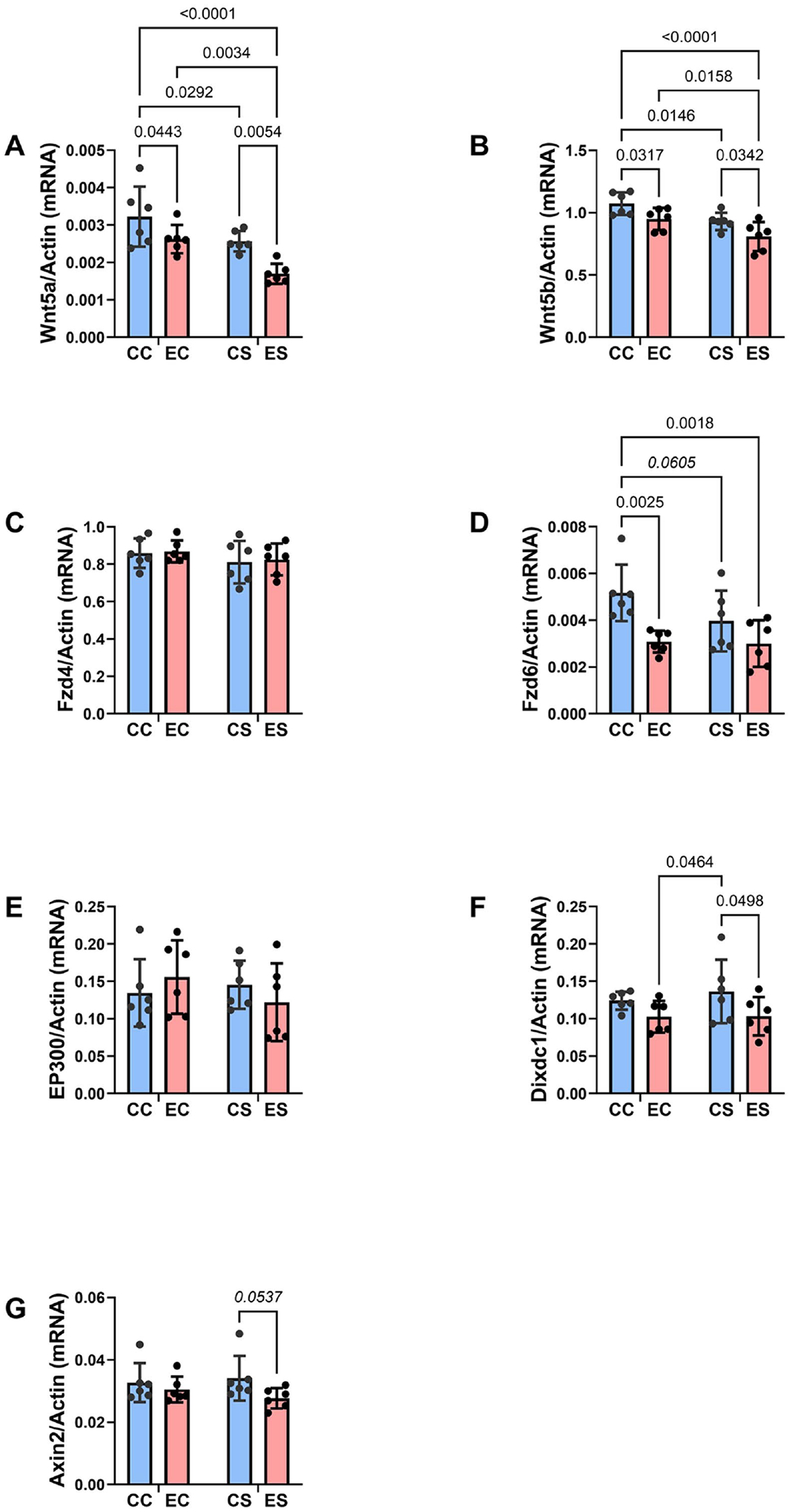
Ethanol and soy effects on wnt signaling networks. RNA was isolated from fresh frozen temporal tissue, and mRNA expression was measured by duplex qRT-PCR analysis with a probe-hydrolysis detection system. *β*-actin was amplified and detected in the same well as the gene of interest (see Methods). Graphs depict relative mRNA abundance (mean ± S.D.) of (A) Wnt5a, (B) Wnt5b, (C) Frizzled 4 (Fzd4), (D) Fzd6, (E) EP300, (F) Dixdc1, and (G) Axin2. Two-way ANOVA tests were used for inter-group comparisons (see Table 6). Significant (p ≤ 0.05) and statistical trend wise (0.05 < p < 0.10; italics) inter-group differences detected with post hoc multiple comparisons tests are displayed within the panels.

Two-way ANOVA tests revealed significant dietary protein effects on Wnt5a and Wnt5b, and significant ethanol effects on Wnt5a, Wnt5b, Fzd6, and Dixdc1 (Table 6). There were no significant or trend-wise dietary protein × ethanol interactive effects on any of the Wnt pathway molecules studied. The corresponding Wnt pathway graphs with post hoc test results revealed significantly reduced expression levels of Wnt5a (Figure 7(A)) and Wnt5b (Figure 7(B)) in EC compared with CC, in ES relative to CS, and in CS and ES relative to their corresponding casein groups. FZD6 immunoreactivity (Figure 7(D)) was significantly reduced in EC versus CC, but not ES relative to CS, and it was reduced in CS (trend-wise) and ES (significant) relative to CC. Dixdc1 immunoreactivity was significantly lower in EC and ES relative to CS (Figure 7(F)), and Axin2 expression was trend-wise reduced in ES relative to CS (Figure 7(G)). In contrast, the mean levels of Fzd4 (Figure 7(C)) and EP300 (Figure 7(E)) were similar across all groups.

### GnRH Pathway mRNA Studies:

**Figure 8.**
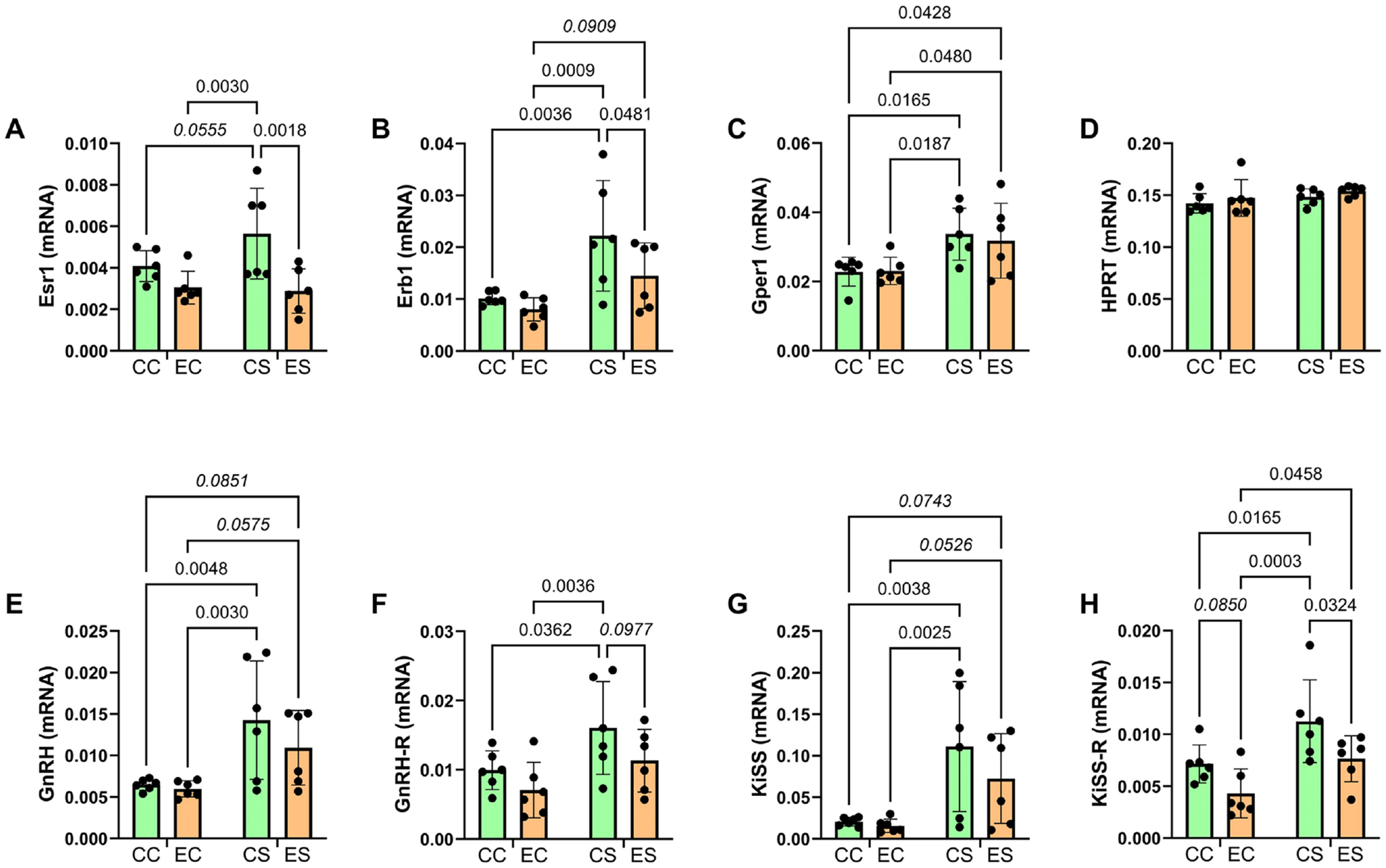
Ethanol and soy effects on GnRH-Related signaling networks. RNA isolated from temporal lobe tissue was used to measure relative mRNA abundance of (A) Esr1, (B) Erb1, (C) Gper1, (E) GnRH, (F) GnRH-R, (G) KiSS, and (H) KiSS-R. (D) HPRT mRNA served as a control for calculating the relative mRNA abundance of specific target genes of interest. Two-way ANOVA tests were used for inter-group comparisons (see Table 7). Significant (p ≤ 0.05) and statistical trend wise (0.05 < p < 0.10; italics) intergroup differences detected with post hoc multiple comparisons tests are displayed within the panels.

Two-way ANOVA tests demonstrated significant dietary soy effects on Erb1, Gper, GnRH, GnRH-R, KiSS1, and KiSS1-R, significant ethanol effects on Esr1 and KiSS1-R, and statistical trend-wise ethanol effects on Erb1 and GnRH-R (Table 7). There were no significant or trend-wise dietary protein × ethanol interactive effects on any of the molecules examined in this pathway. The graphs with post hoc test results revealed significantly higher levels of Esr1 (Figure 8(A)) and Erb1 (Figure 8(B)) in CS relative to the CC, EC, and ES groups. Gper1 (Figure 8(C)), GnRH (Figure 8(E)), KiSS1 (Figure 8(G)), and KiSS1-R (Figure 8(H)) mRNA levels were all significantly higher in both CS and ES relative to CC and EC. GnRH-R mRNA levels were significantly higher in CS than CC and EC, and trend-wise, they increased relative to ES (Figure 8(F)). Specific ethanol effects marked by reductions in mRNA were observed between CS and ES with respect to Esr1 (Figure 8(A)), Erb1 (Figure 8(B)), and KiSS-R (Figure 8(H)), and trend-wise reductions were observed for GnRH (CS versus ES) and KiSS-R (CC versus EC) (Figure 8(H)). HPRT mRNA levels were similar among the four groups (Figure 8(D)).

## 4. Discussion

This manuscript reports the effects of gestational ethanol exposure and dietary soy intervention on postnatal temporal lobe development and function in an experimental model of FASD. In previous reports with the same model, we observed phenotypic craniofacial features of FASD in the late fetal/early postnatal period and correlated those effects with impairments in placentation [55]. Remarkably, co-administration of dietary soy to the pregnant dams largely prevented FASD and its associated abnormalities in placentation [3] [56]. The present work extends the overall effort by determining if dietary soy restricted to the gestation period would be adequate for normalizing postnatal neurodevelopment and function. The most significant findings with translational relevance were that 1) neurodevelopmental abnormalities resulting from gestational alcohol exposure persisted despite withdrawal at delivery, and 2) dietary soy administration only in the gestation period was beneficial in largely reducing the adverse neurobehavioral effects of FASD. It is noteworthy that, unlike previous studies of adult alcohol exposure models [57] [58] and humans with alcohol use disorder [59]–[61] in which cessation of alcohol exposure diminished alcohol-related brain pathologies, in our model, postnatal cessation of chronic alcohol exposure was associated with persistent deficits in spatial learning and memory, lower brain weight, and histopathological abnormalities in the CA1 region of the hippocampus and temporal lobe white matter myelin integrity. The reduced mean brain weight in the EC group reflects the long-term effects of prenatal alcohol exposure on brain growth, as reported previously for experimental models [50] [51] [62] and humans [63]–[65]. These findings suggest that some aspects of alcohol-related brain degeneration are not readily reversed by abstinence alone, but the use of dietary soy or possibly pharmaceutical insulin sensitizer/antioxidant agents may offer significant therapeutic potential for normalizing brain growth and temporal lobe/hippocampal structure and functions needed for learning and memory. Additional ongoing studies will assess responses to graded concentrations of dietary soy to determine the minimum requirement for preserving neurodevelopment in FASD models.

The brain samples were obtained 35 days after cessation of both the ethanol and soy exposures. Molecular assays were used to delineate the underlying mechanisms of neurocognitive impairment via signaling pathways known to mediate normal CNS development and function. At the same time, the strategy helped to inform on how dietary soy mediates its preventive or therapeutic effects in FASD. The analyses focused on the insulin/IGF/IRS/Akt, Notch, and Wnt signaling pathways because previous studies showed their substantial vulnerabilities to alcohol exposure in the brain, including in development [34] [44] [49] [51] [66] [67]. In addition, we evaluated the effects on the GnRH pathway due to its relevance to neurodevelopment and cognitive-behavioral function in adolescence [45] [46] [68].

In contrast to earlier findings in which chronic gestational exposure to alcohol was shown to inhibit insulin and IGF-1 receptor expression and signaling (tyrosine phosphorylation) through insulin receptor substrate molecules, the findings in the present study revealed no significant inhibitory effects of ethanol on these upstream molecules. Only a trend-effect of reduced pS-IRS-1 in EC relative to CC was observed, which would have been protective since Serine phosphorylation of IRS-1 inhibits signaling [53]. The rescue effects of gestational dietary soy on spatial learning and memory and temporal lobe histology were not associated with positive directional shifts in insulin receptor, IGF-1 receptor, or IRS-1 signaling. Although the relative levels of tyrosine phosphorylated IGF-1 were trend-wise increased by soy, irrespective of ethanol exposure, IGF-1R and pY-IGF-1R were significantly reduced, suggesting that overall signaling through IGF-1R was not enhanced by soy. Similarly, IRS-1 expression was reduced in the CS and ES samples, indicating that signaling downstream via IRS-1 was not improved by dietary soy. Although incomplete, the cessation of alcohol exposure at birth was likely sufficient to reduce but not abolish long-term effects.

Downstream signaling through Akt, GSK-3*β*, and P70S6K was largely unaffected by prenatal ethanol in the absence of soy. The trend-wise reduction in pS9-GSK-3*β* in EC and significant reduction in ES relative to CC suggest the adverse effects of prenatal ethanol on GSK-3*β* activation persisted to some degree, despite cessation of ethanol exposure at birth or gestational dietary soy feeding of the dams. The significance of the relative reductions in pS-Akt/total Akt in EC, CS, and ES is unclear but not likely to account for the inter-group differences in neurobehavioral function, brain size, or temporal lobe histology. Phosphorylation of PRAS40 enhances neuronal survival and increases mTOR activation to inhibit autophagy [69]–[71]. Considering the evidence that dietary soy was neuroprotective, its inhibitory effects on PRAS40 and pT-PRAS40 expression would seem paradoxical. However, in previous studies, a naturally occurring flavonoid was observed to be neuroprotective and provide antioxidant effects but also produce dual inhibitory effects on PI3K/Akt and mTOR with associated reductions in p-PRAS40 and p-P70S6K [72].

In the brain, Notch pathways regulate neurodevelopment, modulating differentiation, migration, survival, and plasticity in neurons [73]. Notch signaling is mediated by the interactions of Notch heterodimeric receptors with cell surface ligands such as Jagged expressed on nearby cells. Subsequent proteolytic cleavages release the Notch intracellular domain, resulting in its translocation to the nucleus where it binds to transcriptional regulators and ultimately leads to increased expression of Hairy-enhancer of Split (HES), HEY, and HES-related protein gene families [73]. Major developmental outcomes of Notch signaling include neurogenesis, gliogenesis, neuronal migration, dendritic and synaptic plasticity, and behavioral functions linked to learning and memory [73] [74].

ASPH is a type 2 transmembrane protein with a C-terminal catalytic domain that hydroxylates aspartyl- and asparaginyl residues in EGF-like domains of Notch and Jagged [5] [54] [75] [76]. Correspondingly, ASPH has demonstrated roles in neuronal migration, plasticity, growth, and survival, as well as glial functions via Notch-pathway activation [6] [10] [12] [77]. HIF-1*α* is a transcription factor that modulates cellular and tissue adaptive responses to hypoxia. HIF-1*α* has dual effects in inducing neuronal cell death in states of severe hypoxic or ischemia, but under mild circumstances, it can be neuroprotective and promote neuronal survival [78]. HIF-1*α* can mediate its effects by activating Notch via physical interactions with the Notch intracellular domain [79]. Finally, FIH, which like ASPH, has hydroxylase catalytic functions, negatively regulates crosstalk between HIF and Notch by asparaginyl hydroxylation of the Notch ICD, inhibiting HIF activation of Notch [80] [81]. High levels of FIH promote differentiation and inhibit immature functions such as neurogenesis [80]. In addition, FIH inhibits HIF-1*α*’s inhibitory effects on energy metabolism and oxidative phosphorylation in mitochondria [82].

The mRNA studies mainly showed significant stimulatory effects of gestational soy on HES-1 in ES relative to EC, and on HIF-1*α* in CS and ES relative to CC and EC. The increased HES-1 mRNA in the ES temporal lobe samples reflects enhanced Notch signaling, which could be important for promoting neurodevelopment. However, the significantly increased HIF-1*α* in both CS and ES suggests the long-term effects of gestational dietary soy include enhanced signaling through HIF-1*α*, which would also serve to promote Notch pathway activation irrespective of ethanol exposure. The findings by ELISA largely confirmed the results obtained by qRT-PCR analysis in that dietary soy enhanced Notch signaling by increasing HES-1 and HIF-1*α* in both CS and ES relative to CC. At the same time, the increased levels of Jagged-1 and HIF-1*α* immunoreactivity in EC relative to CC could reflect responses to oxidative stress resulting from prior chronic gestational ethanol exposure [6] [83]. In contrast to previous studies of experimental FASD in which ASPH expression and Notch pathway signaling were found to be impaired during the period of chronic ethanol exposure [44], inhibitory effects on these pathways were not observed in EC relative to CC. This suggests that prenatal ethanol-associated impairments in ASPH expression [84] [85] and Notch signaling in the temporal lobes can be at least partly resolved by cessation of ethanol exposure at birth. However, enhanced signaling via HES-1/HIF-1*α* in the CS and ES samples suggests that long-term sustained effects of gestational dietary soy, irrespective of prenatal alcohol exposure, contribute to the improvements in cognitive performance in the acquisition phases of learning and memory on the MWM tests.

Wingless-related integration site (Wnt) genes encode ligands with diverse biological functions and are critical for development, including in the brain [15]. Wnt/*β*-catenin signaling has important roles in neurodevelopment and is adversely impacted by chronic alcohol exposures [44]. Our studies were focused on Wnt5a, which supports neurite outgrowth and synapse formation in immature neurons [86] [87] and is a critical regulator of neurogenesis [88], and Wnt5b, which is related to Wnt5a and needed for cell migration, proliferation, and differentiation, and has critical roles in neurodevelopment [89], but signals through non-canonical Wnt which is *β*-catenin-independent. Frizzled 4 (Fzd4) receptor regulates canonical pathway molecules and has functional roles in the blood-retinal and blood-brain barriers. Fzd6, a non-canonical pathway receptor critical for Wnt signaling via ligand-binding, mediates important functions during development, including synapse formation, synaptic plasticity, and neuronal circuitry needed for diverse CNS functions [90]. Fzd6 also inhibits the canonical pathway, reducing *β*-catenin target gene expression via TCF/LEF transcription factor phosphorylation [91]. The histone acetyltransferase EP300 interacts with erythropoietin receptors and supports neuroprotective and neuro-regenerative functions [92]. In addition, EP300 suppresses hippocampal and canonical Wnt pathways [93]. Disheveled-Axin domain containing-1 (Dixcd1) is a critical canonical pathway regulator of neurodevelopment with roles in neuronal migration [94]. Axin2 is expressed in immature brain oligodendrocyte progenitor cells in white matter and has a critical role in remyelination [95].

The EC- and ES-associated significant reductions in Wnt5a, Wnt5b, and Fzd6 mRNA indicate that chronic prenatal alcohol exposures can have long-lasting inhibitory effects on both canonical (Wnt5a) and non-canonical (Wnt5b, Fzd6) Wnt pathways in the brain. However, the absence of any dietary soy-specific enhancement of Wnt signaling molecule expression suggests that the rescue/normalization effects of soy were not mediated by bolstering Wnt pathways. Instead, alternative mechanisms must be implicated.

GnRH signaling plays a critical role in regulating reproductive functions in mammals via its actions on G-protein coupled receptors with attendant synthesis and secretion of gonadotropin hormones [96]. The potential adverse effects of prenatal alcohol exposure on GnRH-related signaling in the brain were investigated because, in addition to its role in pubescent development and function of the hypothalamic-pituitary axis [45], emerging evidence suggests broader significance in relation to cognition, neuronal plasticity, behavior, neurodevelopment, and function [22] [45] [46]. Prenatal alcohol exposures were shown to delay puberty onset and alter GnRH neuronal morphology [97]. In prepubertal female rats, alcohol administration reduces GnRH secretion due to inhibition of glial transforming growth factor beta1 (TGF*β*1) responsiveness [98]. Furthermore, independent studies have shown that dietary soy and related isoflavones enhance signaling through estrogen receptors and G protein-coupled estrogen receptors like Gper1 in the developing brain and have antioxidant activity [99]. Regarding growth and plasticity, isoflavones can increase signaling through mitogen-activated protein kinases and phosphatidylinosidide-3-kinase (PI3K) and thereby activate Akt and inhibit GSK-3*β* [99]. Important effects of isoflavone stimulation include improved spatial learning and memory, dendritic arborization, and synaptogenesis in the brain [99]. Of further relevance to the present work is that soy isoflavones target both the hippocampus and cerebral cortex, promoting neurogenesis, dendritic arborization, and insulin receptor substrate activation [99].

The studies herein showed that in ES, prenatal ethanol exposure inhibited or blunted the dietary soy-stimulated increases in temporal lobe Esr1 and Erb1 expression observed in CS brains. In contrast, Gper1 expression was similarly upregulated in CS and ES relative to CC and EC. The observed stimulatory effects of soy/isoflavones on estrogen receptor and Gper1 in CS are consistent with previous reports [99]. The inhibitory effects of binge alcohol consumption on estrogen receptor mRNA expression and function have also been reported in a mouse model [100]. Increased Gper expression has been linked to improvements in memory and hippocampal plasticity [101], corresponding with the enhanced spatial learning observed in the CS and ES rats on Trial Days 1 and 2 of MWM testing. However, the absence of significant reductions in Esr1, Erb1, or Gper1 in the EC relative to CC group suggests that the EC-related impairments in MWM performance were not mediated by the inhibition of estrogen receptor signaling. At the same time, the absence of soy-stimulated increases in Esr1 and Erb1 expression in ES relative to CS did not adversely impact neurobehavioral function given their similar MWM performances.

GnRH/GnRH-R and KiSS/KiSS-R have established roles in mediating neuronal plasticity [22] [45] [46]. Dietary soy administered to the pregnant dams significantly increased temporal lobe expression of these molecules, irrespective of chronic prenatal ethanol exposure. Like Gper1, the overall robust increases in mRNA expression correlate with enhanced MWM performance. However, the degrees of increased GnRH-R and KiSS-R expression in ES were trend-wise (GnRH-R) or significantly (KiSS-R) lower than in CS, corresponding with the significant but suboptimal improvement in MWM performance (Figure 1(F)), and the known inhibitory effects of alcohol on KiSS-1 expression [41] [102]. Altogether, the analyses of GnRH signaling mechanisms suggest that the cessation of ethanol exposure in the early postnatal period abolished the inhibitory effects previously reported, but the stimulatory effects of gestational soy on these pathways in early adolescence were largely sustained in both CS and ES, although the robustness of the responses was reduced by prior ethanol exposure. We hypothesize that the soy-induced stimulation of GnRH pathways accounts for the increased brain weights, normalized or nearly normalized hippocampal structure, and enhanced performance on MWM tests in the ES group. Importantly, dietary soy, even if restricted to the period of gestation, may help reduce FASD effects mediated by early to mid-gestation alcohol exposures.

The dietary soy rescue approach emerged from earlier studies demonstrating the therapeutic effects of PPAR agonists on neurodevelopmental, neurodegenerative, and cognitive-behavioral impairments in experimental models of brain insulin resistance, including ARBD [25] [26] [103] [104]. However, the realization that PPAR agonist drugs might adversely impact the developing brain and pregnant women, encouraged us to consider alternative strategies that incorporated natural products, especially nutrient-rich foods. Dietary soy was predicted to address this need because soy isoflavones have potent PPAR agonist effects, enhancing insulin responsiveness and energy and lipid metabolism [105] [106]. Regarding the present work, soy isoflavones have been shown to activate Notch [107] and regulate cell migration, maturation, morphogenesis and metabolism in the developing brain [99]. Moreover, soybean isoflavones, via their interactions with ER and GPER have critical roles in astrocytic function, synaptogenesis, and dendritic arborization needed for learning and memory [108]. Correspondingly, PPAR agonists modulate Notch signaling by altering the expression of Notch ligands or its downstream target molecules [109], and they stimulate GnRH, possibly via the hypothalamic/pituitary axis [110]. Therefore, isoflavones contained within dietary soy likely mediate supportive effects on neurodevelopment and function in FASD via their interactions with and stimulation of Notch and GNRH signaling molecules, mimicking the effects of PPAR agonists. However, additional studies are needed to determine if the full spectrum of FASD might be prevented by extending dietary soy supplementation into the postnatal period and perhaps through the critical phases of adolescent brain development. Furthermore, studies of cognitive-behavioral function in rats that survive into adulthood would provide additional valuable information concerning the long-term benefits of dietary soy interventions in FASD.

## 5. Conclusions

- Dietary soy prevented alcohol-related reduction in brain weight and significantly increased spatial learning and memory in ethanol-exposed offspring.
- Postnatal cessation of alcohol exposure largely prevented or resolved the impairments in CNS insulin/IGF-1/IRS1/Akt, Notch, and Wnt signaling that is associated with ongoing ethanol exposures.
- Major broad effects of gestational dietary soy include significantly increased expression of multiple genes that mediate gonadotropin signaling, which is known to have important roles in neurodevelopment during adolescence.
- The findings highlight the need to experimentally evaluate the potential benefits of Notch and GnRH pathway therapeutic targeting to prevent long-term adverse neurodevelopmental effects of prenatal alcohol exposure.

## Supplementary Material

1

## Figures and Tables

**Table 1.** Rat primers for qRT-PCR Array-GnRH Pathway Primers.

Rat primer	Sequence 5’ - 3’	Gene ID	Position	Amplicon bp
R-Esr1 forward1	ACTGTCTCAGCTCTTGACTTCTAC	NM_001433730.1	103 – 126	
R-Esr1 reverse1	TCATGGTCATGGTCAGTGGC		232 – 251	149
R-Erb1 forward	TCGTTCTGGACAGGGATGAG	AB190769.1	1183 – 1202	
R-Erb1 reverse	CTGGAGTTGAGGAGGATCATGG		1309 – 1330	148
R-Gper1 forward	TCTTCGCAGTGGTCCTTGTC	NM_133573.2	827 – 846	
R-Gper1 reverse	GTCAAGGGGTAGGCATGACG		940 – 959	133
R-GnRH forward1	CAGCACTGGTCCTATGGGTT	M15527.1	181 – 200	
R-GnRH reverse1	TGGTGCTGGCAAACTTACCTC		111 – 131	90
R-GnRHR forward2	TCACACGAGTCCTTCATCAGG	U00935.1	1389 – 1409	
R-GnRHR reverse2	TAGAGTTCTCAGCCGTGCTC		1455 – 1474	86
R-KISS1 forward2	TCTCCTCTGTGTGGCCTCTTT	JX139031.1	240 – 260	
R-KISS1 reverse2	TGCCAGGCATTAACGAGTTCC		330 – 350	111
R-KISS1R forward1	TGCTGGGAGACTTCATGTGC	NM_023992.2	500 – 519	
R-KISS1R reverse1	AACACAGTCACGTACCAGCG		592 – 611	112
R-HPRT forward	AGACAGCGGCAAGTTGAATC	NM_012583.2	725 – 744	
R-HPRT reverse	ATGGCCACAGGACTAGAACG		786 – 805	81
R-Actin forward	TTGCTGACAGGATGCAGAAG	NM_031144	1004 – 1023	
R-Actin reverse	ACATCTGCTGGAAGGTGGAC		1125 – 1144	141

Forward and reverse rat (R) primer pairs for PCR amplification of cDNA targets generated by 1^st^ strand synthesis reactions using random hexamers. The Gene ID, initial nucleotide primer binding position of the primers, and the sizes of the amplicons resulting from PCR amplification.

**Table 2.** Effects of dietary protein source and ethanol on body and brain weights.

Index	CC	EC	CS	ES	ANOVA
Dam body weight-GD21 Mean ± S.D. (# Samples)	283.2 ± 24.73 N = 4	281.1 ± 21.75 N = 4	297.9 ± 20.74 N = 4	280.4 ± 16.72 N = 4	N.S.
Offspring birth weight-P0 Mean ± S.D. (# Samples)	6.96 ± 0.39 N = 9	7.10 ± 0.37 N = 13	7.08 ± 0.58 N = 10	6.88 ± 0.79 N = 15	N.S.
Offspring weight-P35 Mean ± S.D.	130.4 ± 10.39 **p = 0.0001**	138.4 ± 14.63 **p = 0.002**	147.1 ± 5.01 **p = 0.04**	151.9 ± 13.82* ---	--- **p = 0.0006**
Offspring brain weight-P35 Mean ± S.D.	1.75 ± 0.018 **p = 0.0097**	1.65 ± 0.02 ---	1.76 ± 0.04 **p = 0.0018**	1.77 ± 0.08 **p < 0.0001**	--- **p < 0.0001**

Inter-group comparisons of maternal body weight at delivery (Gestation Day-GD), pup birth weight on postnatal Day 0 (P0), off-springs’ body and brain weights at P35, the experimental endpoint. All weights are in grams. (grams). Data were analyzed by ANOVA. Significant post hoc test results corresponding to mean weight differences are highlighted with bold font. Significant inter-group differences (p ≤ 0.05) detected by repeated measures multiple comparisons post hoc tests are indicated. Abbreviations: CC = Control Casein; EC = Ethanol-Casein; CS = Control Soy; ES = Ethanol Soy.

**Table 3.** INSULIN/IGF-1, IRS1-Akt PATHWAY-ANOVA TEST RESULTS

Molecule	Diet F Ratio	p-Value	Ethanol F Ratio	p-value	Diet × Ethanol F Ratio	p-Value
Insulin R	2.54	N.S.	0.009	N.S.	0.141	N.S.
IGF-1R	**9.144**	**0.0067**	0.299	N.S.	1.502	N.S.
IRS-1	**10.63**	**0.0039**	0.810	N.S.	0.002	N.S.
Akt	0.127	N.S.	0.245	N.S.	0.091	N.S.
GSK-3*β*	0.835	N.S.	0.193	N.S.	0.083	N.S.
PRAS40	**10.85**	**0.0036**	1.766	N.S.	2.421	N.S.
P70S6K	2.667	N.S.	1.191	N.S.	0.082	N.S.
pY-Insulin R	2.504	N.S.	0.081	N.S.	1.582	N.S.
pY-IGF-1R	**7.860**	**0.011**	2.067	N.S.	2.501	N.S.
pS-IRS-1	0.273	N.S.	1.613	N.S.	2.462	N.S.
pS-Akt	*3.197*	*0.089*	0.004	N.S.	1.552	N.S.
pS-GSK-3*β*	2.212	N.S.	*3.482*	*0.077*	0.478	N.S.
pT-PRAS40	**26.24**	**<0.0001**	**5.636**	**0.028**	*4.082*	*0.057*
pTpS-P70S6K	**5.565**	**0.028**	0.110	N.S.	0.661	N.S.
pY/Insulin R	0.084	N.S.	0.0396	N.S.	0.361	N.S.
pY/IGF-1 R	**6.254**	**0.021**	0.063	N.S.	0.079	N.S.
pS/IRS-1	1.02	N.S.	0.51	N.S.	2.763	N.S.
pS/Akt	5.647	0.027	2.27	N.S.	2.346	N.S.
p/T-GSK-3b	0.223	N.S.	1.453	N.S.	0.559	N.S.
pT/PRAS40	**30.75**	**<0.0001**	**5.265**	**0.033**	2.634	N.S.
pTpS/P70S6K	1.180	N.S.	0.078	N.S.	0.155	N.S.

Immunoreactivity was measured with a 7-Plex Akt Pathway magnetic bead-based panel. The Table lists the two-way ANOVA test results (F-Ratios and P-Values). For all tests: Diet factor (Soy or Casein as the protein source), Ethanol Factor, and Diet × Ethanol Interaction, DFn, DFd (1, 28). Bold font highlights significant results (p < 0.05). Italics corresponds to a statistical trend (0.05 < p < 0.10). N.S. = not significant. N = 6 rats/group. See Figure 4 and Figure 5 for corresponding graphs and post hoc test results. Abbreviations: R = receptor; IGF = insulin-like growth factor; IRS = insulin receptor substrate; GSK-3*β* = glycogen synthase kinase-3*β*; PRAS40 = proline-rich Akt substrate of 40 kDa; P70S6K = 70-kDa ribosomal protein S6 kinase; pY = tyrosine phosphorylated; pS = serine phosphorylated; pT = threonine phosphorylated.

**Table 4.** ASPH-NOTCH NETWORK-(mRNA)-ANOVA test results.

Molecule	Diet F Ratio	p-Value	Ethanol F Ratio	p-Value	Diet × Ethanol F Ratio	p-Value
ASPH	0.029	N.S.	0.761	N.S.	1.115	N.S.
NOTCH 1	0.688	N.S.	0.130	N.S.	1.562	N.S.
JAGGED 1	0.094	N.S.	**0.047**	N.S.	5.025	**0.036**
HES 1	4.50	**0.046**	*0.059*	N.S.	1.866	N.S.
HIF-1*α*	12.34	**0.0022**	0.662	N.S.	0.088	N.S.
FIH	0.466	N.S.	0.274	N.S.	0.006	N.S.

The mRNA levels were measured with a duplex PCR (Taqman-based) assay with results normalized to actin measured in the same samples. The Table lists the two-way ANOVA test results (F-Ratios and P-Values). For all tests: Diet factor, Ethanol Factor, and Diet × Ethanol Interaction DFn, DFd (1, 28). Bold font highlights significant results (p ≤ 0.05). Statistical trendwise differences (0.05p < 0.10) are italicized. N.S. = not significant. N = 6 rats/group. See Supplementary Figure 1 for corresponding graphs and post hoc test results. Abbreviations: ASPH = aspartyl-asparaginyl-*β*-hydroxylase; HES1 = hairy and enhancer of split-1; HIF-1*α* = hypoxia inducible factor 1*α*; FIH = factor inhibiting hypoxia-inducible factor 1.

**Table 5.** ASPH-NOTCH NETWORK-IMMUNOREACTIVITY-ANOVA test results.

Molecule	Diet F Ratio	p-Value	Ethanol F Ratio	p-Value	Diet × Ethanol F Ratio	p-Value
ASPH-A85G6	2.777	N.S.	*2.962*	*0.10*	2.370	N.S.
ASPH-A85E6	2.393	N.S.	0.268	N.S.	5.108	0.035
NOTCH 1	0.607	N.S.	0.157	N.S.	0.001	N.S.
JAGGED 1	2.513	N.S.	1.111	N.S.	**7.277**	**0.0139**
HES 1	**6.316**	**0.021**	**4.315**	**0.05**	0.355	N.S.
HIF-1*α*	**7.628**	**0.012**	*3.254*	*0.086*	1.747	N.S.

Immunoreactivity was measured in temporal lobe tissue by duplex ELISAs with results normalized to RPLPO measured in the same samples. The Table lists the two-way ANOVA test results. For all tests: Diet factor, Ethanol Factor, and Diet × Ethanol Interaction, DFn, DFd (1, 28). Bold font highlights significant results (p ≤ 0.05). Statistical trend wise differences (0.05p < 0.10) are italicized. N.S. = not significant. N = 6 rats/group. See Figure 6 for corresponding graphs and post hoc test results. Abbreviations: ASPH = aspartyl-asparaginyl-*β*-hydroxylase (measured with two different monoclonal antibodies; HES1 = hairy and enhancer of split-1; HIF-1*α* = hypoxia inducible factor 1*α*; FIH = factor inhibiting hypoxia-inducible factor 1.

**Table 6.** Wnt PATHWAY (mRNA)-ANOVA test results.

Molecule	Diet F Ratio	p-Value	Ethanol F Ratio	p-Value	Diet × Ethanol F Ratio	p-Value
Wnt 5a	**16.07**	**0.0007**	**13.85**	**0.0013**	0.472	N.S.
Wnt 5b	**14.11**	**0.0012**	**10.50**	**0.0041**	0.0006	N.S.
Fzd 4	1.667	N.S.	0.115	N.S.	0.005	N.S.
Fzd 6	2.26	N.S.	**12.78**	**0.0019**	1.716	N.S.
EP300	0.382	N.S.	0.0036	N.S.	1.47	N.S.
Dixdc1	0.322	N.S.	**5.933**	**0.024**	0.268	N.S.
Axin2	0.098	N.S.	*3.799*	*0.065*	0.903	N.S.

mRNA levels were measured with a duplex PCR (Taqman-based) assay with results normalized to Actin. The Table lists the two-way ANOVA test results (F-Ratios and P-Values). For all tests: Diet factor, Ethanol Factor, and Diet × Ethanol Interaction, DFn, DFd (1, 28). Bold font highlights significant results (p < 0.05). Statistical trend wise differences (0.05p < 0.10) are italicized. N.S. = not statistically significant. N = 6 rats/group. See Figure 7 for corresponding graphs and post hoc test results. Abbreviations: Fzd = Frizzled; EP300 = E1A binding protein p300; Dixdc1 = Disheveled-Axin domain containing-1.

**Table 7.** GnRH PATHWAY (mRNA)-ANOVA test results.

Molecule	Diet F Ratio	p-Value	Ethanol F Ratio	p-Value	Diet × Ethanol F Ratio	p-Value
Esr1	1.651	N.S.	**12.17**	**0.0023**	2.531	N.S.
Erb1	**12.89**	**0.0018**	*3.605*	*0.072*	1.163	N.S.
Gper1	**11.15**	**0.0033**	0.079	N.S.	0.1299	N.S.
GnRH	**13.44**	**0.0015**	1.218	N.S.	0.6653	N.S.
GnRH-R	**7.241**	**0.014**	*3.892*	*0.062*	0.235	N.S.
KiSS1	**14.25**	**0.0012**	1.237	N.S.	0.741	N.S.
KiSS1-R	**11.27**	**0.003**	**8.452**	**0.0087**	0.1188	N.S.
HPRT	2.079	N.S.	1.432	N.S.	0.001	N.S.

mRNA levels were measured with a custom targeted PCR array with results normalized to HPRT. The Table lists the two-way ANOVA test results (F-Ratios and P-Values). For all tests: Diet factor, Ethanol Factor, and Diet × Ethanol Interaction, DFn, DFd (1, 28). Bold font highlights significant results (p ≤ 0.05). Statistical trend-wise differences (0.05p < 0.10) are italicized. N.S. = not significant. N = 6 rats/group. See Figure 8 for corresponding graphs and post hoc test results. Abbreviations: Esr1 = Estrogen Receptor 1; Erb1 = Estrogen Receptor b1; Gper = G-Protein Coupled Estrogen Receptor; GnRH = Gonadotropin releasing hormone; GnRH-R = Gonadotropin releasing hormone receptor; KiSS1 = Kisspeptin 1; KiSS1-R = KiSS-1 receptor; HPRT1 = hypoxanthine phosphoribosyltransferase.

## Data Availability

The data underlying this article will be shared at a reasonable request by the corresponding author.
