# Supplementary material for: Dietary Soy Preserves Cognitive Function in Experimental Fetal Alcohol Spectrum Disorder: Role of Increased Signaling through Notch and Gonadotropin Releasing Hormone Networks
Source: J Behav Brain Sci. Author manuscript; Available in PMC 2025 Aug 20. (PMC12364082; doi:10.4236/jbbs.2025.152002)
Supplement: 1 [file NIHMS2096427-supplement-1.pdf]

Appendix

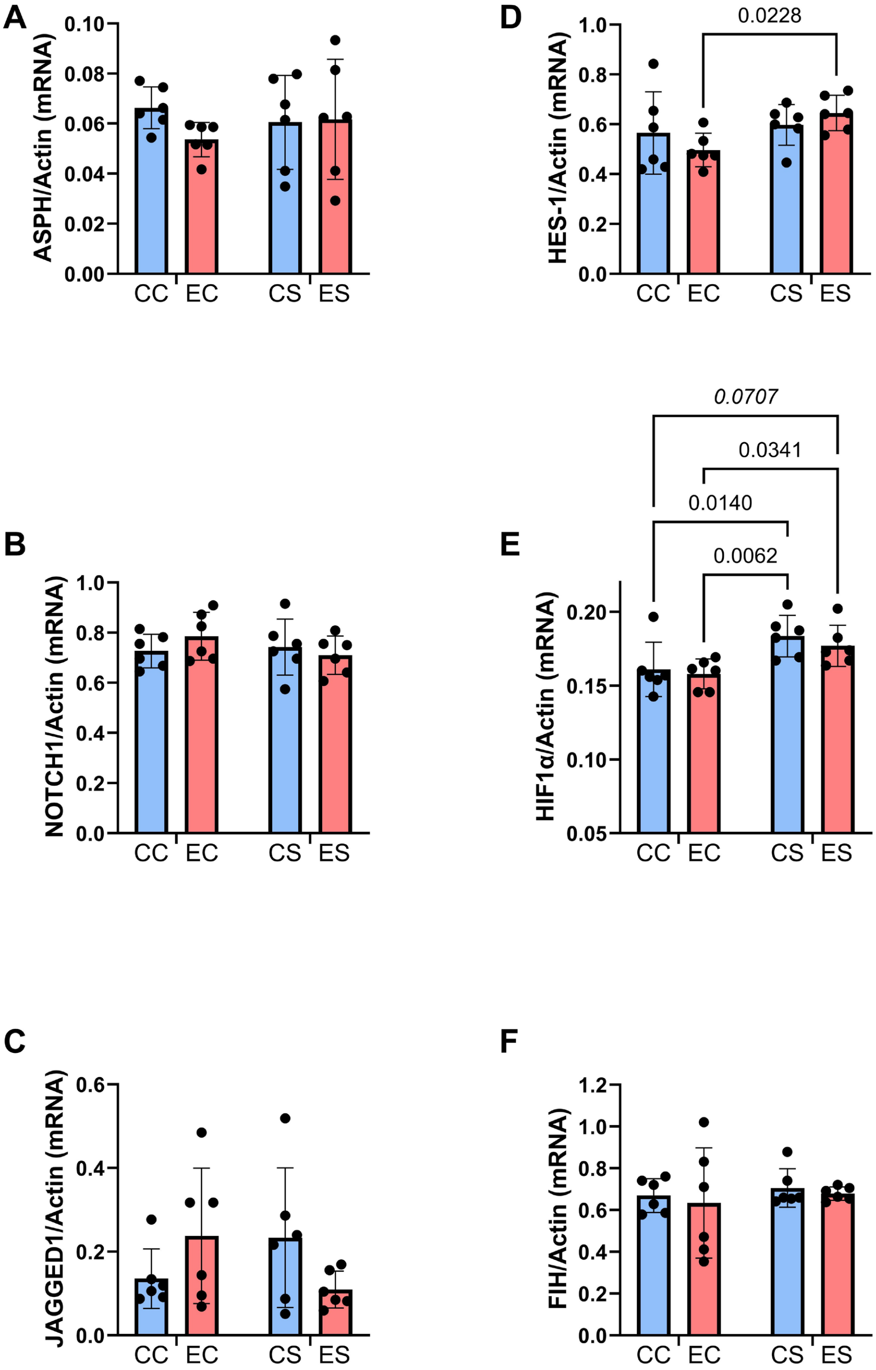

Supplementary Figure 1. Ethanol and Dietary Soy Effects on ASPH and Notch Networks. Gene expression was measured by duplex qRT-PCR analysis with a probe-hydrolysis detection system. The β-actin housekeeping gene was simultaneously amplified and detected in the same well as the gene of interest (see Methods). Graphs depict relative mRNA abundance (mean ± S.D.) of (A) ASPH, (B) Notch1, (C) Jagged1, (D) HES1, (E) HIF-1α, and (F) FIH. Two-way ANOVA made inter-group comparisons (see Table 4) with post-hoc Tukey tests. Significant ($p \leq 0.05$) inter-group differences detected with post hoc tests are displayed within the panels.
